# Total Nut, Tree Nut, and Peanut Consumption and Metabolic Status in Southern Italian Adults

**DOI:** 10.3390/ijerph18041847

**Published:** 2021-02-14

**Authors:** Agnieszka Micek, Justyna Godos, Achille Cernigliaro, Raffaele Ivan Cincione, Silvio Buscemi, Massimo Libra, Fabio Galvano, Giuseppe Grosso

**Affiliations:** 1 Department of Nursing Management and Epidemiology Nursing, Faculty of Health Sciences, Jagiellonian University Medical College, 31-501 Krakow, Poland; 2 Department of Biomedical and Biotechnological Sciences, University of Catania, 95123 Catania, Italy; justyna.godos@gmail.com (J.G.); m.libra@unict.it (M.L.); fgalvano@unict.it (F.G.); giuseppe.grosso@unict.it (G.G.); 3 Department of Health Service and Epidemiological Observatory, Health Authority Sicily Region, 90145 Palermo, Italy; achille.cernigliaro@regione.sicilia.it; 4 Department of Clinical and Experimental Medicine, University of Foggia, 71122 Foggia, Italy; ivan.cincione@unifg.it; 5 Biomedical Department of Internal and Specialist Medicine (DIBIMIS), University of Palermo, 90123 Palermo, Italy; silvio.buscemi@unipa.it; 6 Research Center for Prevention, Diagnosis and Treatment of Cancer, University of Catania, 95123 Catania, Italy

**Keywords:** tree nut, peanut, almonds, hypertension, type-2 diabetes, dyslipidemia, Mediterranean diet, polyphenols, phenolic acids, cohort

## Abstract

Background: Nut consumption has been associated with cardio-metabolic health benefits. However, studies conducted in the Southern Italian population, where adherence to the Mediterranean diet has been reported being relatively high, are rather scarce. The aim of this study was to test the association between consumption of total and specific types of nuts and metabolic status among adults living in Sicily, Southern Italy. Methods: Demographic and dietary characteristics of 2044 adults living in Southern Italy were analyzed. Multivariate logistic regression analyses were performed to calculate odds ratios (ORs) and 95% confidence intervals (CIs) of the association between nut consumption and metabolic status adjusting for potential confounding factors. Results: The energy-adjusted model revealed that higher nut intake was inversely associated with occurrence of hypertension, type-2 diabetes, and dyslipidemia. However, the association did not remain significant for the latter after adjusting for the main background characteristics, while an inverse association was stably confirmed for hypertension (OR = 0.61, 95% CI: 0.46–0.80 and OR = 0.44, 95% CI: 0.26–0.74, respectively) even after adjusting for adherence to the Mediterranean diet. Among individual nut types, most of the associations were null except for higher almond intake, which was inversely associated with occurrence of hypertension (OR = 0.70, 95% CI: 0.49–0.99). Conclusions: Higher nut consumption is associated with overall better metabolic status in individuals living in the Mediterranean area.

## 1. Introduction

The result of a pioneering, large, prospective cohort study (the Adventist Health Study) published in the early 1990s suggested that high consumption of nuts provides protection against coronary heart disease risk [1]. Since then, the rapidly growing body of literature has demonstrated the potential beneficial influence of nuts on human health, showing that a diet enriched with nuts may reduce the incidence of some cardiometabolic diseases and mortality [2].

Nuts that grow on trees are called tree nuts and are dry, hard-shelled, pod-shaped fruits consisting of one edible seed. The most consumed nuts in Europe are almonds (*Prunus amigdalis*), hazelnuts (*Corylus avellana*), walnuts (*Juglans regia*), pistachios (*Pistachia vera),* and chestnuts (*Castanea sativa*) [3]. Peanuts (*Arachis hypogaea*), also known as the groundnut, is a legume crop widely grown in the tropics and subtropics and, by far, the most consumed nut worldwide [4]. Nuts are nutrient-rich foods containing meaningful amounts of proteins, fiber, unsaturated fatty acids, minerals, vitamins, and other bioactive antioxidant compounds, such as polyphenols and phytosterols [5].

Nuts have numerous unique nutritional attributes, such as richness in vegetal protein and being poor in sodium but high in other essential minerals (such as iron, calcium, copper, phosphorus, magnesium, zinc, and selenium), that could be recommended as a source of protein for vegans giving up eating meat and fish, pregnant women, and elderly individuals [6]. Moreover, adding nuts as a component of healthy dietary patterns has been advocated as a dietary strategy of facilitating control weight maintenance and cardioprotection [7].

In recent decades, the role of nutritional habits, including intake of nuts, in the prevention of metabolic disorders have been increasingly investigated [8,9]. The metabolic abnormalities, such as central adiposity, dyslipidemia, hypertension, hyperglycemia, hyperinsulinemia, and type-2 diabetes, synergistically contribute to the increased risk of morbidity and mortality worldwide [10]. Summary of evidence suggests a potential beneficial influence of nuts on the metabolic profile, as regular nut consumers have a tendency to be leaner, as well as to gain body weight more slowly over time [11]. Moreover, inclusion of nuts within the context of healthy dietary patterns, including (but not limited to) the Mediterranean diet, has proven interesting against metabolic outcomes [12]. However, the relation between nut consumption on metabolic abnormalities in Italian adults, especially with regard to individual types of nuts and adherence to the Mediterranean diet, has never been investigated. Thus, the aim of this study was to examine the association between total and individual types of nuts and metabolic outcomes in a sample of Southern Italian adults.

## 2. Materials and Methods

### 2.1. Study Population

The Mediterranean healthy Eating, Aging, and Lifestyle (MEAL) study is an observational study conducted in Mediterranean island of Sicily aimed to investigate the association between traditional nutritional and lifestyle habits and non-communicable diseases. Details of the study protocol are published elsewhere [13]. Individuals were randomly selected in the main districts of the city of Catania, Southern Italy. The enrollment and data collection was performed between 2014 and 2015 through the registered records of local general practitioners stratified by sex and 10-year age groups. The theoretical sample size was set at 1500 individuals to provide a specific relative precision of 5% (Type I error, 0.05, Type II error, 0.10), taking into account an anticipated 70% participation rate. Out of 2405 individuals invited, the final sample size was 2044 participants (response rate of 85%). The study enrollment process is shown in Supplementary Figure S1. All participants were informed about the aims of the study and provided a written informed consent. All the study procedures were carried out in accordance with the Declaration of Helsinki (1989) of the World Medical Association and approved by the Institutional Review Board (or Ethics Committee) of University of Catania (protocol code 802/23 December 2014).

### 2.2. Data Collection

An electronic data collection was performed by a face-to-face assisted personal interview, using tablet computers. In order to visualize the response options, participants were provided with a paper copy of the questionnaire. However, final answers were registered directly by the interviewer. The background characteristics included sex, age at recruitment, highest educational degree achieved, and occupation (specifies the character of the most important employment during the year before the investigation) or last occupation before retirement. Occupational status was categorized as: (i) unemployed, (ii) low (unskilled workers), (iii) medium (partially skilled workers), and (iv) high (skilled workers). Educational status was categorized as: (i) low (primary/secondary), (ii) medium (high school), and (iii) high (university). Physical activity was evaluated with the International Physical Activity Questionnaires (IPAQ) [14], which included a set of questionnaires (five domains) investigating the time spent being physically active in the last seven days. Based on the IPAQ guidelines, the final score allowed us to categorize the physical activity level as (i) low, (ii) moderate, and (iii) high. Smoking status was categorized as: (i) non-smoker, (ii) ex-smoker, and (iii) current smoker, while alcohol consumption was categorized as: (i) none, (ii) moderate drinker (0.1–12 g/d), and (iii) regular drinker (>12 g/d). Anthropometric measurements were performed according to standardized methods [15]. Height was measured to the nearest 0.5 cm without shoes, with the back square against the wall tape, eyes looking straight ahead, with a right-angle triangle resting on the scalp and against the wall. The body mass index (BMI) was calculated, and patients were categorized as under/normal weight (BMI < 25 kg/m^2^), overweight (BMI 25 to 29.9 kg/m^2^), and obese (BMI ≥ 30 kg/m^2^).

### 2.3. Dietary Assessment

The dietary assessment was performed by the administration of two food frequency questionnaires (FFQ, a long and a short version) that have been previously tested for validity and reliability for the Sicilian population [16,17]. Intake of seasonal foods referred to consumption during the period in which the food was available and then adjusted by its proportional intake in one year. The identification of the food intake, the energy content, and the macro-, micro-nutrient and mineral intake were obtained through a comparison with food composition tables of the Italian Research Center for Foods and Nutrition [18]. The process of the estimation of habitual polyphenol intake has been retrieved from the Phenol-Explorer database, as previously described in detail [19]. The food consumption was calculated (in g or ml) by following the standard portion sizes used in the study and then converted in 24-h intake. Then, the databases was searched to retrieve mean content values for macronutrients, micronutrients, and polyphenols contained in the foods obtained. The nutrient and polyphenol intake from each food was calculated by multiplying the content by the daily consumption of each food adjusted for total energy intake (kcal/d) using the residual method [20]. FFQs with unreliable intakes (<1000 or >6000 kcal/d) were excluded from the analyses (*n* = 198), leaving a total of 1846 individuals included in the analysis. The variables of interest were total and individual types of nuts, including chestnuts, hazelnuts, almonds, walnuts, and peanuts.

Adherence to the Mediterranean diet was assessed using a literature-based score not including nut consumption among its criteria [21]. The score was built by reviewing existing studies on the association between adherence to the Mediterranean diet and various health outcomes, weighting all the median (or mean) values for the sample size of each study population, and then calculating a mean value of all the weighted medians to identify an “average” serving size for each food group included in the existing studies. Two standard deviations were used to determine three different categories of consumption for each food group [22]. Positive points were assigned for consumption of food groups characterizing a Mediterranean dietary diet, such as fruit, vegetables, legumes, cereals, fish, and olive oil. Negative points were assigned for excess consumption of food groups not representing it, such as meat and dairy products. Moderate alcohol intake was deemed as optimal for higher adherence. The final adherence score comprises nine food categories with a score ranging from 0 point (lowest adherence) to 18 points (highest adherence) and individuals grouped in tertiles and categorized as low, medium, and high adherence to the Mediterranean diet.

### 2.4. Metabolic Outcomes

Arterial blood pressure was measured at the end of the physical examination with the subject in a sitting position and at least 5 min at rest. Blood pressure measurements were taken three times at the right arm, which is relaxed and well supported by a table, with an angle of 45 degrees from the trunk. A mean of the last two measurements was considered for inclusion in the database. Information from measurements was integrated with general practitioners computerized records, as a specialist diagnoses patients with disease in order to obtain drugs. Patients have been considered hypertensive when average systolic/diastolic blood pressure levels were greater or equal to 140/90 mmHg, taking anti-hypertensive medications, or being previously diagnosed of hypertension. Patients were considered diabetic or dyslipidemic whether previously diagnosed of diabetes and hypercholesterolemia/hypertriglyceridemia, respectively.

### 2.5. Statistical Analysis

Frequencies were expressed as absolute numbers and percentages, while continuous variables were expressed as means and standard deviations. Individuals were grouped based on total nut intake (median point used as a cut-off) and distribution of background characteristics were compared between groups. In order to test whether nut consumption was related to other dietary variables, including polyphenol intake, a comparison of median (and standard errors) macronutrients and micronutrients, total polyphenols, and individual nut type by total nut intake was also conducted. Differences were tested with a Chi-square test for categorical variables, Student t-test for continuous variables distributed normally, and Mann-Whitney U-test for variables distributed abnormally.

Energy-adjusted multivariate logistic regression models were used to calculate odds ratios (ORs) and 95% confidence intervals (CIs) for association between total and individual type of nut intake and metabolic outcomes. A multivariate model adjusted for all other background characteristics (BMI, physical activity, educational status, occupational status, smoking status) was also performed in order to test whether the observed associations were independent from the previously mentioned variables. An additional model further adjusting for adherence to the Mediterranean diet was used to test whether the retrieved associations were independent of the overall quality of the diet.

All reported *P*-values were based on two-sided tests, and a significance level was 5%. SPSS 21 (SPSS Inc., Chicago, IL, USA) software was used for all the statistical analysis.

## 3. Results

The median intake of total nuts in the study population was 11.6 g/d. The mean intake of total nuts in the groups categorized as “high nut intake” (above the median) was 39.7 g/d, while those in “low nut intake” (under the median) was 4.3 g/d. Only 137 individuals (7.4%) reported not consuming nuts at all. The distribution of background characteristics by total nut intake is shown in Table 1. Comparison between groups (tested with a Chi-square test) revealed that there was a significantly higher proportion of older participants (21.9% vs. 16.4% older than 65 years old, *p* = 0.004) of a low occupational level (17.4% vs. 12.8%, *p* = 0.022) among those individuals consuming less nuts compared to higher consumers. However, no clear trends were observed and all variables examined had comparable distribution across categories (Table 1). However, a significant difference was observed on the level of adherence to the Mediterranean diet, as there was a higher proportion of high nut consumers among those individuals with medium and high adherence to the dietary pattern (overall 52% vs. 38.9%, *p* <0.001) (Table 1).

Micronutrient, macronutrient, total polyphenol, mineral, and individual type of nut intake by total nut intake is shown in Table 2. The comparison of mean intake through a Student t-test or a Mann-Whitney U-test showed that those individuals consuming more nuts had higher energy intake and higher intake of most macronutrients and micronutrients as well as total polyphenols (Table 2). Moreover, most minerals were higher in diets including high intake of nuts. Total nut intake was also not affected by the contribution of a single type of nut, as none of the comparisons between nut type was significant as well (Table 2).

Table 3 shows the association between total and individual type of nut intake and metabolic outcomes. The energy-adjusted model revealed that higher nut intake was associated with lower odds of hypertension, Type 2 diabetes, and dyslipidemia compared to lower nut intake. However, the association did not remain significant for the latter after adjusting for the main background characteristics, while an inverse association was stably confirmed for hypertension (OR = 0.61, 95% CI: 0.46–0.80 and OR = 0.44, 95% CI: 0.26–0.74, respectively) even after adjusting for the level of adherence to the Mediterranean diet. Among individual nut types, most of the associations remained null, with the exception of higher almond intake associated with lower odds of hypertension (OR = 0.70, 95% CI: 0.49–0.99).

## 4. Discussion

In the present study, the association between nut intake and metabolic outcomes has been investigated in a sample of adults living in the Mediterranean area. While an inverse association between total nut intake and hypertension and Type 2 diabetes has been found, we failed to find a relation between individual type of nut intake and the investigated outcomes with the exception for almond intake, which was inversely associated with occurrence of hypertension. Notably, despite higher nut intake being more common in individuals more adherent to the Mediterranean diet, adjustment for this variable did not significantly change the strength and direction of the retrieved associations.

Nut consumption has been generally associated with cardiovascular benefits, but results toward metabolic outcomes are not univocal in the scientific literature: in fact, despite acute feeding studies have shown favorable effects on fasting blood glucose [23], no association between nut consumption and the risk of Type 2 diabetes mellitus was observed in meta-analyses of prospective studies [24]. Moreover, nuts have been shown to affect blood lipids despite the effects being stronger among individuals with blood lipid abnormalities [25]. Up to date, the strongest evidence from epidemiological studies relates nut consumption with lower risk of hypertension [26]. Moreover, meta-analyses of clinical trials investigating short-term effects showed an improvement in endothelial function (measured as flow-mediated dilation) [27] and lowering blood pressure effects in subjects without Type 2 diabetes mellitus [28]. Finally, studies conducted in the Mediterranean area demonstrated beneficial effects of higher nut intake toward incidence of metabolic syndrome and cardiometabolic risk factors [29,30] as well as all-cause mortality [31].

The mechanisms explaining the potential benefits of nut consumption toward metabolic health are common to all outcomes and are substantially represented by modulation of inflammation and oxidative stress [32]. Meta-analyses of clinical trials showed a significant effect in reducing leptin and intercellular adhesion molecule-1 (ICAM-1) levels, despite no significant effect being found for other inflammatory biomarkers [33,34]. Nuts are a source of vegetable essential fatty acids and amino acids, fiber, antioxidant vitamins, phytochemicals, and minerals, which all contribute to the previously mentioned effects [5]. Higher intake of monounsaturated fatty acids (MUFA) has been associated with a decrease in postprandial activation of factor VII in response to meals rich in fats and reduction in the ex vivo activation of platelets [35]. Poly-unsaturated fatty acids (PUFA) are precursors of anti-inflammatory prostaglandins and leukotrienes, which are able to reduce serum concentrations of the vasoconstrictor thromboxane 2. Thus, both MUFA and PUFA may play a role in reducing platelet aggregation, coagulation, and thrombosis [36]. Nuts are also rich in L-arginine, which is a semi-essential amino acid that has been reported to improve insulin resistance, decrease advanced glycation end products (AGEs) formation, increase nitric oxide (NO production), and decreasing levels of angiotensin II and oxidative stress, with improved endothelial cell function and decreased peripheral vascular resistance [37,38]. Antioxidant vitamins, such as group B vitamin, folate, tocopherols, and phenolic compounds, including a variety of flavonoids and phenolic acids, have been shown to possess a range of bioactive properties, such as antioxidant, anti-inflammatory, anti-proliferative, hypocholesterolemic effects, and anti-hypertensive effects [39,40]. Emerging evidence suggests that the interaction between polyphenols and gut microbiota may be responsible for immune system regulation and reduction in local and systemic inflammation [41,42]. Minerals including magnesium, potassium, and calcium, which play a role in regulating blood pressure by producing NO, regulating prostacyclin, and calcium channel blockade, by reducing the extracellular fluid volume modulating the activity of the renin-angiotensin system, and by inhibiting the parathyroid hormone, which induces hypertension [43].

In the present study, we found that consumption of almonds was associated with lower odds of hypertension independently of the other nuts. Current evidence suggests that whole almond consumption may play a role in reducing inflammation, oxidative stress, and dysmetabolism occurring in metabolic syndrome, despite the specific function of almond polyphenol metabolites in the prevention of chronic disease still emerging [44]. Whether differences in health benefits between nut type occur need to be further investigated. Currently, the most common tree nuts used in intervention studies have been walnuts, which are likely among the most consumed nuts worldwide [45]. Concerning almonds, in line with our results, a number of clinical trials have found significant improvements in fasting blood glucose and diastolic blood pressure significantly associated with almond consumption despite the cut-off point identified in the meta-analysis being 42.5 g/d [46,47], which is higher than those reported in our study.

With special regard to polyphenol content, it has been recently reported that nut consumption may be a major contributor to total polyphenol intake in Southern European individuals, with phenolic acids being the polyphenol class mostly represented in nuts [19]. Moreover, investigating the type of nut consumed by a population may lead to important differences in terms of the group of polyphenols. For instance, walnuts and pistachios provide higher content (more than double) of total phenols than hazelnuts and almonds. However, almonds and pistachios have the highest content in flavonoids, with differences in specific classes (almonds have more flavonols, pistachios have more flavan-3-ols and anthocyanins) [48]. In this study, we showed that individuals having higher intake of total nuts consumed nearly double the number of total polyphenols than those having lower intake. Nuts have been reported to contribute to a large proportion of total polyphenols and up to 90% of hydroxybenzoic acids to the daily diet of Southern European individuals. While dietary hydroxybenzoic acids are generally provided by tea in Northeastern European populations, in the southern ones, tea is not consumed as much due to different cultural traditions [49]. Thus, nuts may represent a valid alternative to tea to integrate diet with important polyphenols not otherwise consumed.

The results of the present study need to be considered in light of some limitations. First, the observational nature of the study does not allow us to assess cause-effect relations, but only associations. Second, the dietary information has been retrieved through an FFQ, which may suffer from recall bias, with overestimation or underestimation of intake of food groups included. Third, nut consumption has been reported to be associated with an overall healthier lifestyle [9]. Thus, although we adjusted for several potential confounding factors, we cannot rule out the possibility of residual confounding. Finally, the confidence intervals for the intake of almonds were close to the null value. Thus, results should be considered with caution.

## 5. Conclusions

In conclusions, higher nut consumption is associated with overall better metabolic status in individuals living in the Mediterranean area. However, whether this is related to an overall healthier lifestyle or specifically affected by a higher intake of nuts in the traditional diet must still be elucidated. Further research exploring the association between nut intake and cardiometabolic health by taking into account the metabolites of their consumption is needed.

## Figures and Tables

**Table 1:** Background characteristics of the study sample by consumption of nuts (cut-off 11.6 g/d referring to median intake). BMI: body mass index

	Low Nut Intake (Mean 4.3 g/d)	High Nut Intake (Mean 39.7 g/d)	*p*-Value
**Sex, *n* (%)**			0.619
Male	379 (41.7)	380 (40.6)	
Female	530 (58.3)	557 (59.4)	
**Age group, *n* (%)**			0.004
<30	173 (19.0)	169 (18.0)	
30–44	243 (26.7)	247 (26.4)	
44–65	294 (32.3)	367 (39.2)	
>65	199 (21.9)	154 (16.4)	
**Educational level, *n* (%)**			0.064
Low	317 (34.9)	346 (36.9)	
Medium	325 (35.8)	361 (38.5)	
High	267 (29.4)	230 (24.5)	
**Occupational level, *n* (%)**			0.022
Unemployed	214 (25.5)	227 (30.9)	
Low	146 (17.4)	94 (12.8)	
Medium	225 (26.8)	198 (27.0)	
High	253 (30.2)	215 (29.3)	
**Smoking status, *n* (%)**			0.497
Non-smoker	552 (60.7)	590 (63.0)	
Current smoker	220 (24.2)	222 (23.7)	
Former smoker	137 (15.1)	125 (13.3)	
**Physical activity level, *n* (%)**			0.345
Sedentary	161 (20.0)	156 (18.6)	
Low	387 (48.0)	432 (51.6)	
Medium	258 (32.0)	249 (29.7)	
High			
**BMI categories, *n* (%)**			0.657
Normal	395 (46.6)	418 (48.0)	
Overweight	306 (36.1)	296 (34.0)	
Obese	146 (17.2)	156 (17.9)	
**Mediterranean diet adherence, *n* (%)**			<0.001
Low	556 (61.2)	450 (48.0)	
Medium	276 (30.4)	383 (40.9)	
High	77 (8.5)	104 (11.1)	
**Health status, *n* (%)**			
Hypertension	487 (53.6)	427 (45.6)	<0.001
Type-2 diabetes	78 (8.6)	61 (6.5)	0.092
Dyslipidemia	174 (19.1)	164 (17.5)	0.363

**Table 2:** Micronutrient, macronutrient, polyphenol, and individual type of nut intake by total nut intake in the study sample.

	Low Nut Intake	High Nut Intake	*p*-Value
Energy intake (kcal/d)	2104.5 (44.2)	2090.8 (44.2)	<0.001
**Macronutrients**			
Carbohydrates (g/d)	343.8 (7.8)	294.7 (7.1)	<0.001
Fiber (g/d)	36.7 (1.2)	31.0 (0.9)	<0.001
Protein (g/d)	82.3 (2.1)	84.8 (2.1)	<0.001
Fat (g/d)	51.6 (1.4)	64.0 (1.4)	<0.001
Cholesterol (mg/d)	155.4 (6.7)	197.3 (6.1)	<0.001
SFA %	19.9 (0.6)	24.9 (0.5)	<0.001
MUFA %	23.3 (0.5)	26.1 (0.5)	<0.001
PUFA %	10.1 (0.2)	11.3 (0.3)	<0.001
Total Omega-3 g (g/d)	1.4 (0.0)	1.2 (0.0)	<0.001
**Micronutrients**			
Vitamin A (Retinol) (mg/d)	724.5 (11.7)	821.9 (15.9)	<0.001
Vitamin C (mg/d)	122.7 (3.2)	141.0 (3.2)	<0.001
Vitamin E (mg/d)	7.2 (0.1)	8.8 (0.1)	<0.001
Vitamin B12 (mg/d)	5.0 (0.2)	6.0 (0.1)	<0.001
Vitamin D (mg/d)	3.5 (0.1)	4.6 (0.2)	<0.001
**Total polyphenols**	514.4 (25.0)	544.7 (19.4)	<0.001
**Minerals**			
Sodium (mg/d)	2617.6 (32.4)	2765.3 (40.5)	<0.001
Potassium (mg/d)	3183.5 (40.5)	3709.3 (48.8)	<0.001
Magnesium (mg/d)	403.4 (11.2)	387.2 (9.6)	<0.001
Selenium (mg/d)	114.0 (3.6)	100.7 (2.9)	0.153
Zinc (mg/d)	12.4 (0.3)	12.4 (0.3)	<0.001
Calcium (mg/d)	665.9 (26.8)	756.7 (21.3)	<0.001
Iron (mg/d)	15.3 (0.5)	15.4 (0.4)	<0.001
**Nut groups**			
Chestnuts (g/d)	4.5 (2.1)	4.5 (3.2)	0.215
Peanuts (g/d)	0.6 (0.2)	0.6 (0.1)	0.103
Pistachios (g/d)	0.3 (0.0)	0.3 (0.0)	0.278
Walnuts (g/d)	0.6 (0.2)	0.6 (0.3)	0.205
Almonds (g/d)	0.6 (0.3)	0.6 (0.2)	0.485
Hazelnuts (g/d)	0.6 (0.3)	0.0 (0.2)	0.957

Data are presented as median (standard errors). Abbreviations: MUFA (monounsaturated fatty acids), PUFA (polyunsaturated fatty acids), SFA (saturated fatty acids).

**Table 3:** Association between nut consumption (highest vs. lowest median intake) and metabolic outcomes in the study sample.

	Hypertension	Type 2 Diabetes	Dyslipidemia
	OR (95% CI) *		
**Total nuts**			
Model 1 ^a^	0.67 (0.56–0.81)	0.33 (0.22–0.49)	0.62 (0.49–0.80)
Model 2 ^b^	0.62 (0.47–0.80)	0.45 (0.26–0.75)	0.78 (0.55–1.10)
Model 3 ^c^	0.61 (0.46–0.80)	0.44 (0.26–0.74)	0.78 (0.55–1.10)
**Chestnuts**			
Model 1 ^a^	0.94 (0.76–1.17)	0.79 (0.52–1.18)	0.76 (0.57–1.01)
Model 2 ^b^	0.95 (0.70–1.29)	0.68 (0.39–1.17)	0.75 (0.50–1.12)
Model 3 ^c^	0.97 (0.71–1.31)	0.67 (0.39–1.17)	0.75 (0.50–1.12)
**Peanuts**			
Model 1 ^a^	0.89 (0.70–1.15)	1.07 (0.67–1.73)	0.72 (0.51–1.00)
Model 2 ^b^	0.92 (0.65–1.29)	1.17 (0.62–2.22)	0.68 (0.43–1.08)
Model 3 ^c^	0.91 (0.64–1.28)	1.16 (0.61–2.20)	0.68 (0.43–1.09)
**Pistachios**			
Model 1 ^a^	0.84 (0.64–1.10)	0.67 (0.39–1.15)	0.85 (0.59–1.22)
Model 2 ^b^	0.87 (0.60–1.26)	0.62 (0.30–1.30)	1.16 (0.71–1.90)
Model 3 ^c^	0.86 (0.59–1.25)	0.63 (0.30–1.30)	1.18 (0.72–1.93)
**Walnuts**			
Model 1 ^a^	1.09 (0.86–1.40)	0.89 (0.56–1.42)	1.22 (0.89–1.67)
Model 2 ^b^	1.17 (0.83–1.65)	0.79 (0.42–1.49)	1.03 (0.66–1.61)
Model 3 ^c^	1.18 (0.83–1.67)	0.84 (0.44–1.60)	0.99 (0.63–1.55)
**Almonds**			
Model 1 ^a^	0.77 (0.61–0.98)	1.31 (0.84–2.04)	1.11 (0.81–1.51)
Model 2 ^b^	0.70 (0.50–1.00)	1.22 (0.66–2.24)	1.23 (0.79–1.92)
Model 3 ^c^	0.70 (0.49–0.99)	1.17 (0.63–2.15)	1.26 (0.80–1.96)
**Hazelnuts**			
Model 1 ^a^	0.96 (0.73–1.26)	0.94 (0.56–1.58)	0.91 (0.64–1.29)
Model 2 ^b^	1.07 (0.73–1.56)	1.35 (0.70–2.60)	1.12 (0.69–1.80)
Model 3 ^c^	1.06 (0.73–1.55)	1.34 (0.70–2.58)	1.12 (0.70–1.81)

* The odds ratios refers to the highest vs. the lowest median intake of nut including: total nuts > 11.6 g/d (mean 39.7 g/d vs. 4.3 g/d), chestnuts > 4.5 g/d (mean 33.7 g/d vs. 2.1 g/d), peanuts > 0.6 g/d (mean 3.3 g/d vs. 0.2 g/d), pistachios > 0.3 g/d (mean 1.6 g/d vs. 0.1 g/d), walnuts > 0.6 g/d (mean 4.5 g/d vs. 0.3 g/d), almonds > 0.6 g/d (mean 4.7 vs. 0.2 g/d), and hazelnuts > 0.6 g/d (mean 3.9 g/d vs. 0.2 g/d). ^a^ Adjusted for total energy intake. ^b^ Adjusted as model 1 plus age, sex, educational and occupational status, smoking status, and physical activity level. ^c^ Adjusted as a model 2 plus adherence to the Mediterranean diet.

## Supplementary Materials

The following are available online at https://www.mdpi.com/1660-4601/18/4/1847/s1. Figure S1. The study enrollment process.

## References

[1] Fraser G.E., Sabaté J., Beeson W.L., Strahan T.M. (1992). A possible protective effect of nut consumption on risk of coronary heart disease. The Adventist Health Study. Arch. Intern. Med..

[2] Martini D., Godos J., Marventano S., Tieri M., Ghelfi F., Titta L., Lafranconi A., Trigueiro H., Gambera A., Alonzo E. (2021). Nut and legume consumption and human health: An umbrella review of observational studies. Int. J. Food Sci. Nutr..

[3] Jenab M., Sabaté J., Slimani N., Ferrari P., Mazuir M., Casagrande C., Deharveng G., Tjønneland A., Olsen A., Overvad K. (2006). Consumption and portion sizes of tree nuts, peanuts and seeds in the European Prospective Investigation into Cancer and Nutrition (EPIC) cohorts from 10 European countries. Br. J. Nutr..

[4] Arya S.S., Salve A.R., Chauhan S. (2016). Peanuts as functional food: A review. J. Food Sci. Technol..

[5] de Souza R.G.M., Schincaglia R.M., Pimentel G.D., Mota J.F. (2017). Nuts and human health outcomes: A systematic review. Nutrients.

[6] Grosso G., Estruch R. (2016). Nut consumption and age-related disease. Maturitas.

[7] Vadivel V., Kunyanga C.N., Biesalski H.K. (2012). Health benefits of nut consumption with special reference to body weight control. Nutrition.

[8] Kim Y., Keogh J., Clifton P.M. (2018). Nuts and Cardio-Metabolic Disease: A Review of Meta-Analyses. Nutrients.

[9] Grosso G., Yang J., Marventano S., Micek A., Galvano F., Kales S.N. (2015). Nut consumption on all-cause, cardiovascular, and cancer mortality risk: A systematic review and meta-analysis of epidemiologic studies. Am. J. Clin. Nutr..

[10] GBD 2019 Risk Factors Collaborators (2020). Global burden of 87 risk factors in 204 countries and territories, 1990–2019: A systematic analysis for the Global Burden of Disease Study 2019. Lancet.

[11] Li H., Li X., Yuan S., Jin Y., Lu J. (2018). Nut consumption and risk of metabolic syndrome and overweight/obesity: A meta-analysis of prospective cohort studies and randomized trials. Nutr. Metab. (Lond.).

[12] Godos J., Zappalà G., Bernardini S., Giambini I., Bes-Rastrollo M., Martinez-Gonzalez M. (2017). Adherence to the Mediterranean diet is inversely associated with metabolic syndrome occurrence: A meta-analysis of observational studies. Int. J. Food Sci. Nutr..

[13] Grosso G., Marventano S., D’Urso M., Mistretta A., Galvano F. (2017). The Mediterranean healthy eating, ageing, and lifestyle (MEAL) study: Rationale and study design. Int. J. Food Sci. Nutr..

[14] Craig C.L., Marshall A.L., Sjöström M., Bauman A.E., Booth M.L., Ainsworth B.E., Pratt M., Ekelund U., Yngve A., Sallis J.F. (2003). International physical activity questionnaire: 12-country reliability and validity. Med. Sci. Sports Exerc..

[15] Mistretta A., Marventano S., Platania A., Godos J., Galvano F., Grosso G. (2017). Metabolic profile of the Mediterranean healthy Eating, Lifestyle and Aging (MEAL) study cohort. Med. J. Nutrition Metab..

[16] Marventano S., Mistretta A., Platania A., Galvano F., Grosso G. (2016). Reliability and relative validity of a food frequency questionnaire for Italian adults living in Sicily, Southern Italy. Int. J. Food Sci. Nutr..

[17] Buscemi S., Rosafio G., Vasto S., Massenti F.M., Grosso G., Galvano F., Rini N., Barile A.M., Maniaci V., Cosentino L. (2015). Validation of a food frequency questionnaire for use in Italian adults living in Sicily. Int. J. Food Sci. Nutr..

[18] Castiglione D., Platania A., Conti A., Falla M., D’Urso M., Marranzano M. (2018). Dietary micronutrient and mineral intake in the mediterranean healthy eating, ageing, and lifestyle (MEAL) study. Antioxidants.

[19] Godos J., Marventano S., Mistretta A., Galvano F., Grosso G. (2017). Dietary sources of polyphenols in the Mediterranean healthy Eating, Aging and Lifestyle (MEAL) study cohort. Int. J. Food Sci. Nutr..

[20] Godos J., Rapisarda G., Marventano S., Galvano F., Mistretta A., Grosso G. (2017). Association between polyphenol intake and adherence to the Mediterranean diet in Sicily, southern Italy. NFS J..

[21] Marventano S., Godos J., Platania A., Galvano F., Mistretta A., Grosso G. (2018). Mediterranean diet adherence in the Mediterranean healthy eating, aging and lifestyle (MEAL) study cohort. Int. J. Food Sci. Nutr..

[22] Sofi F., Dinu M., Pagliai G., Marcucci R., Casini A. (2017). Validation of a literature-based adherence score to Mediterranean diet: The MEDI-LITE score. Int. J. Food Sci. Nutr..

[23] Blanco Mejia S., Kendall C.W., Viguiliouk E., Augustin L.S., Ha V., Cozma A.I., Mirrahimi A., Maroleanu A., Chiavaroli L., Leiter L.A. (2014). Effect of tree nuts on metabolic syndrome criteria: A systematic review and meta-analysis of randomised controlled trials. BMJ Open.

[24] Schwingshackl L., Hoffmann G., Lampousi A.-M., Knüppel S., Iqbal K., Schwedhelm C., Bechthold A., Schlesinger S., Boeing H. (2017). Food groups and risk of type 2 diabetes mellitus: A systematic review and meta-analysis of prospective studies. Eur. J. Epidemiol..

[25] Altamimi M., Zidan S., Badrasawi M. (2020). Effect of tree nuts consumption on serum lipid profile in hyperlipidemic individuals: A systematic review. Nutr. Metab. Insights.

[26] Schwingshackl L., Schwedhelm C., Hoffmann G., Knüppel S., Iqbal K., Andriolo V., Bechthold A., Schlesinger S., Boeing H. (2017). Food Groups and Risk of Hypertension: A Systematic Review and Dose-Response Meta-Analysis of Prospective Studies. Adv. Nutr..

[27] Neale E.P., Tapsell L.C., Guan V., Batterham M.J. (2017). The effect of nut consumption on markers of inflammation and endothelial function: A systematic review and meta-analysis of randomised controlled trials. BMJ Open.

[28] Mohammadifard N., Salehi-Abargouei A., Salas-Salvadó J., Guasch-Ferré M., Humphries K., Sarrafzadegan N. (2015). The effect of tree nut, peanut, and soy nut consumption on blood pressure: A systematic review and meta-analysis of randomized controlled clinical trials. Am. J. Clin. Nutr..

[29] Fernández-Montero A., Bes-Rastrollo M., Beunza J.J., Barrio-Lopez M.T., de la Fuente-Arrillaga C., Moreno-Galarraga L., Martínez-González M.A. (2013). Nut consumption and incidence of metabolic syndrome after 6-year follow-up: The SUN (Seguimiento Universidad de Navarra, University of Navarra Follow-up) cohort. Public Health Nutr..

[30] Ibarrola-Jurado N., Bulló M., Guasch-Ferré M., Ros E., Martínez-González M.A., Corella D., Fiol M., Wärnberg J., Estruch R., Román P. (2013). Cross-sectional assessment of nut consumption and obesity, metabolic syndrome and other cardiometabolic risk factors: The PREDIMED study. PLoS ONE.

[31] Bonaccio M., Di Castelnuovo A., De Curtis A., Costanzo S., Bracone F., Persichillo M., Donati M.B., de Gaetano G., Iacoviello L. (2015). Nut consumption is inversely associated with both cancer and total mortality in a Mediterranean population: Prospective results from the Moli-sani study. Br. J. Nutr..

[32] López-Uriarte P., Bulló M., Casas-Agustench P., Babio N., Salas-Salvadó J. (2009). Nuts and oxidation: A systematic review. Nutr. Rev..

[33] Mazidi M., Rezaie P., Ferns G.A., Gao H.-K. (2016). Impact of different types of tree nut, peanut, and soy nut consumption on serum C-reactive protein (CRP): A systematic review and meta-analysis of randomized controlled clinical trials. Medicine (Baltim.).

[34] Xiao Y., Xia J., Ke Y., Cheng J., Yuan J., Wu S., Lv Z., Huang S., Kim J.H., Wong S.Y.-S. (2018). Effects of nut consumption on selected inflammatory markers: A systematic review and meta-analysis of randomized controlled trials. Nutrition.

[35] Kelly C.M., Smith R.D., Williams C.M. (2001). Dietary monounsaturated fatty acids and haemostasis. Proc. Nutr. Soc..

[36] DiNicolantonio J.J., OKeefe J. (2019). Importance of maintaining a low omega-6/omega-3 ratio for reducing platelet aggregation, coagulation and thrombosis. Open Heart.

[37] Vasdev S., Gill V. (2008). The antihypertensive effect of arginine. Int. J. Angiol..

[38] Smeets E.T.H.C., Mensink R.P., Joris P.J. (2020). Effects of tree nut and groundnut consumption compared with those of l-arginine supplementation on fasting and postprandial flow-mediated vasodilation: Meta-analysis of human randomized controlled trials. Clin. Nutr..

[39] Bolling B.W., McKay D.L., Blumberg J.B. (2010). The phytochemical composition and antioxidant actions of tree nuts. Asia Pac. J. Clin. Nutr..

[40] Godos J., Sinatra D., Blanco I., Mulè S., La Verde M., Marranzano M. (2017). Association between Dietary Phenolic Acids and Hypertension in a Mediterranean Cohort. Nutrients.

[41] Salvucci E. (2019). The human-microbiome superorganism and its modulation to restore health. Int. J. Food Sci. Nutr..

[42] Mena P., Bresciani L. (2020). Dietary fibre modifies gut microbiota: What’s the role of (poly)phenols?. Int. J. Food Sci. Nutr..

[43] Casas-Agustench P., López-Uriarte P., Bulló M., Ros E., Cabré-Vila J.J., Salas-Salvadó J. (2011). Effects of one serving of mixed nuts on serum lipids, insulin resistance and inflammatory markers in patients with the metabolic syndrome. Nutr. Metab. Cardiovasc. Dis..

[44] Bolling B.W. (2017). Almond polyphenols: Methods of analysis, contribution to food quality, and health promotion. Comp. Rev. Food Sci. Food Safety.

[45] Vinson J.A., Cai Y. (2012). Nuts, especially walnuts, have both antioxidant quantity and efficacy and exhibit significant potential health benefits. Food Funct..

[46] Lee-Bravatti M.A., Wang J., Avendano E.E., King L., Johnson E.J., Raman G. (2019). Almond Consumption and Risk Factors for Cardiovascular Disease: A Systematic Review and Meta-analysis of Randomized Controlled Trials. Adv. Nutr..

[47] Eslampour E., Asbaghi O., Hadi A., Abedi S., Ghaedi E., Lazaridi A.-V., Miraghajani M. (2020). The effect of almond intake on blood pressure: A systematic review and meta-analysis of randomized controlled trials. Complement. Ther. Med..

[48] Bolling B.W., Chen C.-Y.O., McKay D.L., Blumberg J.B. (2011). Tree nut phytochemicals: Composition, antioxidant capacity, bioactivity, impact factors. A systematic review of almonds, Brazils, cashews, hazelnuts, macadamias, pecans, pine nuts, pistachios and walnuts. Nutr. Res. Rev..

[49] Platania A., Castiglione D., Sinatra D., Urso M.D., Marranzano M. (2018). Fluid Intake and Beverage Consumption Description and Their Association with Dietary Vitamins and Antioxidant Compounds in Italian Adults from the Mediterranean Healthy Eating, Aging and Lifestyles (MEAL) Study. Antioxidants.

