# Supplementary material for: Total Nut, Tree Nut, and Peanut Consumption and Metabolic Status in Southern Italian Adults
Source: Int J Environ Res Public Health. 2021 Feb 14;18(4):1847. doi: 10.3390/ijerph18041847 (PMC7918537; doi:10.3390/ijerph18041847)

International Journal of
Environmental Research and Public Health

MDPI

Article

Total Nut, Tree Nut and Peanut Consumption and Metabolic Status in Southern Italian Adults

Agnieszka Micek [1,*], Justyna Godos [2], Achille Cernigliaro [3], Raffaele Ivan Cincione [4], Silvio Buscemi [5], Massimo Libra [2,6], Fabio Galvano [2] and Giuseppe Grosso [2]

Supplementary Figure S1. The study enrollment process.

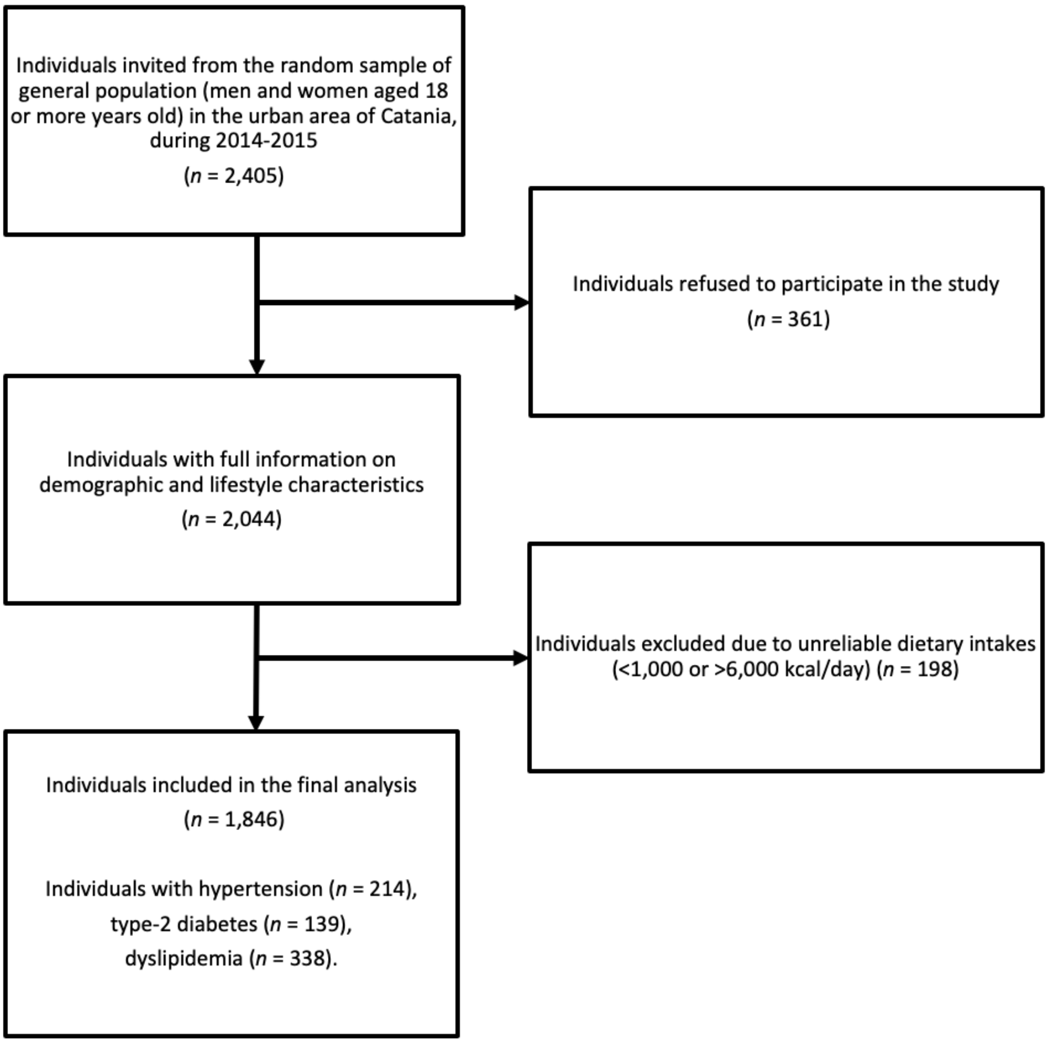

Supplement: Supplementary file 1 [file ijerph-18-01847-s001.pdf]
